# The potential of herbal extracts to inhibit SARS-CoV-2: a pilot study

**DOI:** 10.1186/s40816-021-00264-6

**Published:** 2021-03-08

**Authors:** Michela Luisa De Pellegrin, Anette Rohrhofer, Philipp Schuster, Barbara Schmidt, Philipp Peterburs, André Gessner

**Affiliations:** 1 Institute of Clinical Microbiology and Hygiene, University Hospital Regensburg, Regensburg, Germany; 2 Institute of Medical Microbiology and Hygiene, University of Regensburg, Regensburg, Germany; 3 Bionorica SE, Kerschensteinerstraße 11–15, 92318 Neumarkt, Germany

**Keywords:** SARS-CoV2, Viral replication, Bronchipret thyme-ivy (BRO TI), Bronchipret thyme-primrose (BRO TP), Imupret (IMU), Sinupret extract (SINx), Tonsipret (TOP)

## Abstract

**Background:**

Herbal medicinal products have a long-standing history of use in the therapy of common respiratory infections. We sought to assess the potential of five validated herbal extracts regarding their ability to restrict SARS-CoV-2 replication *in vitro*: Bronchipret thyme-ivy (BRO TI), Bronchipret thyme-primrose (BRO TP), Imupret (IMU), Sinupret extract (SINx) and Tonsipret (TOP).

**Methods:**

Vero cells were incubated with different concentrations of herbal extracts and infected with SARS-CoV-2 for 48 hours. The inhibition of viral replication was assessed by determination of the viral RNA load in the cell culture supernatant using quantitative polymerase chain reaction (qPCR).

**Results:**

SARS-CoV-2 RNA load was reduced by non-cytotoxic concentrations of BRO-TP (up to approximately 1,000-fold) and, to a lesser extent, IMU and TOP (approximately 10-fold).

**Conclusions:**

Some herbal extracts showed a promising *in vitro* effectiveness against SARS-CoV-2, suggesting an antiviral potential of herbal medicinal products. The potential of herbal medicines to restrict SARS-CoV-2 and to treat COVID-19 should be investigated further in a clinical setting.

## Introduction

Coronavirus disease 2019 (COVID-19) is an ongoing pandemic and public health emergency of international concern, caused by severe acute respiratory syndrome coronavirus 2 (SARS-CoV-2). As of 10 February 2020, there have been more than 107 million confirmed infections and more than 2.34 million confirmed deaths worldwide [1].

COVID-19 is primarily a respiratory disease, sometimes leading to severe or fatal pulmonary inflammation and organ failure due to a cytokine storm. Yet, the disease mostly presents with mild to moderate flu-like symptoms such as fever, cough, myalgia as well as taste and smell disturbances [2, 3]. Global research activities are directed towards safe and effective vaccines [4] as well as pharmacological treatments [5]. Pharmacological agents may be directed specifically against a viral or cellular target structure or may involve the unspecific alleviation of the symptom burden that is associated with COVID-19. Overall, there is an urgent need for efficacious COVID-19 therapeutics with little side effects.

Herbal medicinal products are an attractive option for the treatment of infectious diseases and represent a relevant source for pharmacologically active compounds, most prominently demonstrated by the Nobel Prize awarded for the discovery of the herbal component artemisinin – an efficient remedy against malaria [6]. A variety of medicinal plants are believed to have promising antiviral capacity [7]. Herbal medicinal products have the potential to interfere with various steps of the viral replication cycle and/or may be able to strengthen healing and regeneration processes by modulating the host’s immune response in a multimodal manner. Furthermore, herbal medicinal products are well-tolerated due to their low rate of adverse reactions. Many herbal components, e.g. flavonoids, terpenoids, polysaccharides or diverse glycosylated metabolites demonstrate potency against respiratory and inflammatory diseases due to either direct anti-viral or anti-inflammatory effects. In herbal extracts, consisting of a multitude of molecular components, various anti-viral actions may be combined to act in an additive or even synergistic manner [8].

Many of the constituents of marketed herbal medicinal products have already been described to have antiviral activity or to stimulate antiviral defence mechanisms [9–11]. The safety and tolerability of Imupret^®^ (BNO-1030) extract, containing marshmallow root, chamomile flowers, horsetail herb, walnut leaves, yarrow herb, oak bark and dandelion herb, in the treatment of acute viral tonsillitis in children was shown in a randomised clinical trial [8, 12]. Imupret^®^ also showed symptomatic benefits in patients infected with Epstein-Barr virus [12] and inhibited the replication of the common respiratory pathogen respiratory syncytial virus (RSV) in cell culture and animal models [13]. Further, there is encouraging evidence for Sinupret^®^ extract (BNO-1016), an extract of gentian root, primrose flower, elder flower, sorrel herb and verbena herb, as an effective adjunctive treatment in acute rhinosinusitis. This extract significantly reduces the acute symptoms and signs of sinusitis, similarly to other mucoactive agents, with an incidence of adverse events comparable to placebo [14–18]. The antiviral activity of Sinupret^®^ against a range of human respiratory viruses including influenza A virus and RSV has been shown using *in vitro* models [19].

These herbal medicinal products have a long-standing history as over-the-counter medicines for treatment of respiratory infections with a beneficial safety profile. Yet, none of these well-established, efficacious and safe herbal medicines has been tested for their action against SARS-CoV-2, the currently most relevant respiratory pathogen. Here, we report on pilot experiments to assess the *in vitro* potential of a variety of herbal extracts to interfere with SARS-CoV-2.

## Methods

### Extracts

The following extracts (Bionorica SE, Neumarkt in der Oberpfalz, Germany) were investigated:

- Bronchipret^®^ thyme-ivy (BRO TI), an extract of thyme herb (*Thymus vulgaris* l. or *Thymus zygis* l.) and ivy leaves (*Hedera helix *l.). BRO TI is a mixture of fluid extracts of thyme herb (extraction solvent: ammonia solution 10 % (m/m) / glycerol (85 %) (m/m) / ethanol 90 % (v/v) / water (1:20:70:109); drug–extract ratio (DER): 1:2–2.5) and ivy leaves (extraction solvent: ethanol 70 % (v/v); DER: 1:1) as contained in Bronchipret^®^ syrup with a thyme/ivy fluid extract ratio of 10:1. In order to minimise ethanol content in the test system the extract mixture was dealcoholised by rotary evaporation to a final ethanol content of 1 % (v/v). To control for loss of volatile ingredients, specific identity tests were performed with the concentrate.
- Bronchipret® thyme-primrose (BRO TP), an extract of thyme herb (*Thymus vulgaris *l. or *Thymus zygis* l.) and primrose root (*Primula veris *l. or *Primula elatior *(l.) Hill). BROTP is a mixture of genuine dry extracts of thyme herb (extraction solvent: ethanol 70 % (v/v); DER: 6–10:1) and primrose root (extraction solvent: ethanol 47 % (v/v); DER 6–7:1) as contained in Bronchipret^®^ TP film-coated tablets without excipients and with a final thyme/primrose dry extract ratio of 2.67:1.
- Imupret^®^ (IMU), 100 g Imupret oral drops contain: 29 g of an ethanolic-aequous extract (extraction solvent: Ethanol 59 Vol.-%) out of Marshmellow root *(Altheae officinalis *l.) 0.4 g, Chamomille flowers (*Matricaria recutita *l.) 0.3 g, Horsetail herb (*Equisetum* *avense* l.) 0.5 g, Walnut leafs (*Juglans regia *l.) 0.4 g, Yarrow herb (*Achillea millefolium* l.) 0.4 g, Oak bark (*Quercus robur *l.) 0.2 g, Dandelion herb (*Taraxacum officinale* F.H. Wiggers) 0.4 g. Totalethanol 19 % (v/v). In order to minimise ethanol content in the test system the extract mixture was dealcoholised (> 0.5 % (v/v)) by rotary evaporation. The content quality of the dealcoholized test item complied with Imupret^®^ oral drops as checked by identity tests and quantitative analysis.
- Sinupret^®^ extract (SINx), combined genuine dry extract (BNO 1011) of gentian root (*Gentiana lutea* l.), primrose flower (*Primula veris *l.), sorrel herb (*Rumex crispus *l.), elder flower (*Sambucus nigra* l.) and verbena herb (*Verbena officinalis *l.) with a ratio of 1:3:3:3:3 (extraction solvent: ethanol 51 % (v/v); DER 3–6:1) as contained in Sinupret^®^ extract coated tablets without excipients.
- Tonsipret^®^ (TOP), homeopathic dilution for tonsillitis tablets containing 37.5 % Dilution Capsicum D3 (*Capsicum annuum* l.), 37.5 % Dilution Guajacum D3 (*Guaiacum officinale *l./ *Guaiacum sanctum* L.) and 25.0 % mother tincture Phytolacca (*Phytolacca americana *l.). In order to minimise ethanol content in the test system the mixture was dealcoholized (> 0.5 % (v/v)) by rotary evaporation. The quality of the dealcoholised test item complied with the corresponding manufacturing stage of the herbal medicinal product Tonsipret^®^ as checked by identity analyses.

Ethanol concentration in the extracts BRO TI, IMU and TOP was adjusted to 0.37 %. The concentration of the extracts BRO TP and SINx was 100 mg/mL. Extracts were centrifuged (3.000× g for 10 min) and sterile-filtered (pore diameter 0.22 µm). Ethanol concentration was subsequently adjusted to 0.37 % with phosphate-buffered saline (PBS) to obtain the stock solution for experiments. For experiments, a 1:10 dilution of the respective stock solution was subjected to serial twofold dilutions until 1:2,560 in Dulbecco’s Modified Eagle’s Medium (DMEM) plus supplements. Solvent control was ethanol at the same concentration as in the samples.

### Cells and viruses

Vero cells were cultured according to standard procedures in DMEM at 5 % CO_2_ and 37°C. For experiments, cells were seeded in 96-well plates.

Cells were infected with SARS-CoV-2, isolate CA, at a multiplicity of infection (MOI) of 0.05 for 24 hours in the presence of one of the herbal extracts (serial twofold dilutions) or solvent control, washed and incubated for another 24 h in the presence of the herbal extracts prior to harvesting of the cell culture supernatants and quantification of viral ribonucleic acid (RNA) by reverse-transcription/real-time quantitative polymerase chain reaction (qPCR) [20] using the StepOnePlus™ system (Thermo Fisher Scientific) with MS2 as internal control.

Toxicity of the extracts was assessed by the 3-(4,5-dimethylthiazol-2-yl)-2,5-diphenyltetrazolium bromide (MTT) assay [21] after incubation of Vero cells in the absence or presence of serial twofold dilutions of the extracts for 48 h. Prior to addition of MTT, cells were washed and incubated in DMEM plus supplements, but without herbal extracts. The half-maximal inhibitory (IC_50_) and half-maximal cytotoxic concentration (CC_50_) were determined using GraphPadPrism, version 8.4.2. Toxicity equal to or above 50 % was considered substantial.

To assess cell-free virus inactivation, a SARS-CoV-2 virus stock dilution (100 µL) containing 4,000 plaque-forming units (PFU) was incubated at room temperature with selected extracts (100 µL) for 15 min or 1 h, incubation in PBS for 1 h served as control. Incubation was stopped by addition of 800 µL of DMEM, followed by titration on Vero cells. In this experiment, wells were washed 2 h post-infection and incubated in the absence of extracts for 48 h. Titres were quantified by determination of the tissue culture infectious dose (TCID_50_, [22]).

### Statistics

Statistics was calculated using log-transformed viral load data in cell culture supernatants, which were compared to 1:2560 using RM one-way ANOVA with Dunnett’s multiple comparison. *P* values less than 0.05 were considered significant.

## Results

The aim of the pilot experiments described here was to assess whether herbal extracts have the potential to considerably reduce SARS-CoV-2 propagation. Vero cells were chosen as a cell culture system since they are permissive to SARS-CoV-2 [23].

First, Vero cells were incubated with serial twofold dilutions of the extracts for 48 h in order to determine whether they affect cell viability. High concentrations of the extracts resulted in dose-dependent toxicity; half-maximal cytotoxic concentrations (CC_50_) between 1:17 (TOP) and 1:174 (BRO TI) were determined. Toxicity of the extracts was also determined in SARS-CoV-2-infected Vero cells, yielding similar results with CC_50_ values ranging between 1:6.75 (TOP) and 1:153 (BRO TI) (Table 1). Cytotoxicity of ≥ 50 % was considered substantial. Hence, no statements on antiviral effects of the extracts are derived at concentrations ≥ CC_50_.

**Table 1:** Cytotoxicity of herbal extracts in Vero cells. Half-maximal cytotoxic concentrations (CC_50_) as determined by MTT assay

	Uninfected cells	Infected cells
BRO TI	1:174	1:153
BRO TP	1:153.5 (651 µg/ml)	1:156.7 (638 µg/ml)
IMU	1:94.6	1:73.8
SINx	1:58.9 (1,698 µg/ml)	1:52.6 (1,901 µg/ml)
TOP	1:17	1:6.75
Solvent (ethanol)	1:6.3	1:1.73

**Fig. 1:**
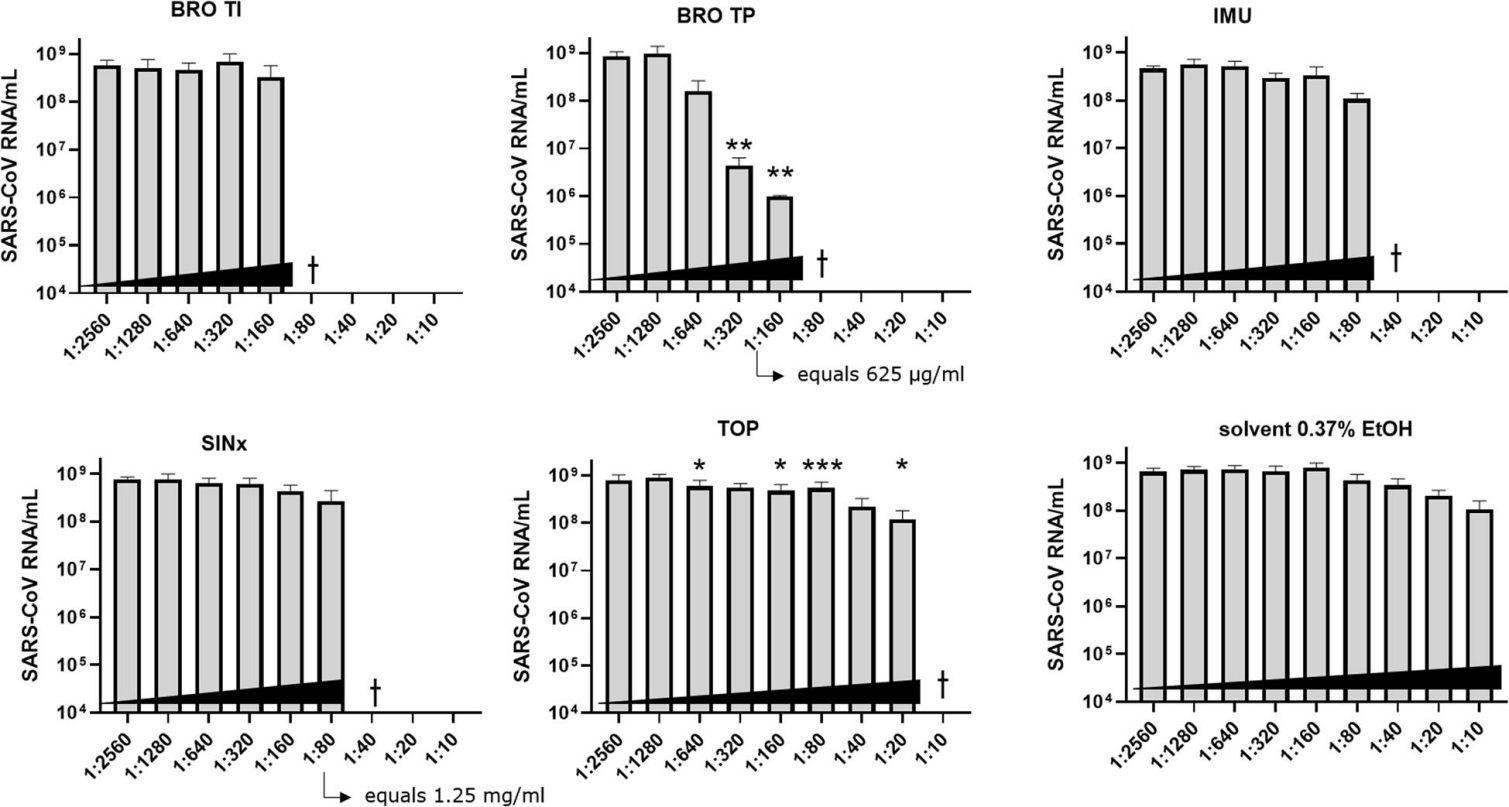
Reduction of SARS-CoV-2 RNA load by herbal extracts. Vero cells were infected with SARS-CoV-2 (MOI 0.05) and treated with serial twofold dilutions of the indicated herbal extracts or solvent (0.37 % ethanol) for 48 h, followed by determination of viral RNA in the supernatant (qPCR). The means of three independent experiments ± SEM are displayed. The dagger symbol (†) indicates concentrations of the extracts ≥ CC_50_. BRO TP and SINx: stock solution was 100 mg/mL, hence concentrations in the assay were 10 mg/mL (dilution 1:10), 5 mg/mL (1:20), 2.5 mg/mL (1:40), 1.25 mg/mL (1:80), 625 µg/mL (1:160), 312.5 µg/mL (1:320), 156.25 µg/mL (1:640), 78 µg/mL (1:1,280) and 39 µg/mL (1:2,560), respectively. Statistics was calculated using log-transformed viral load data in cell culture supernatants, which were compared to 1:2560 using RM one-way ANOVA with Dunnett’s multiple comparison. P values less than 0.05 were considered significant

In order to assess the antiviral capacity of the extracts at non-cytotoxic concentrations, Vero cells were infected with SARS-CoV-2 in the presence of serial twofold dilutions of the extracts or solvent control. At 48 h post-infection, the viral RNA load in the supernatant was determined by real-time qPCR (Fig. 1). The extracts BRO TI and SINx did not considerably reduce the viral RNA load. Reductions in viral RNA load by approximately one order of magnitude were observed for TOP and IMU at the highest non-toxic concentration (< CC_50_). TOP and IMU each reduced the viral RNA load by up to 87 %, similar to the viral load reduction observed with the solvent (ethanol) at the lowest dilution. Strikingly however, BRO TP reduced the SARS-CoV-2 RNA load in a concentration-dependent manner by up to three orders of magnitude (1,000-fold) within the non-cytotoxic range, which suggests considerable antiviral capacity (Fig. 1).

To assess whether the antiviral capacity of BRO TP, in comparison to IMU and TOP was owing to a direct inactivation of the virus, SARS-CoV-2 was incubated with the respective extract for up to 1 h. This treatment reduced viral titres in a time-dependent manner by up to 38 % in case of BRO TP, up to 59 % in case of IMU and up to 65 % in case of TOP compared to incubation for 1 h with PBS (Table 2). This might indicate a potential of herbal extracts to act in a virucidal manner, but does not explain the strong antiviral activity of BRO TP when cultivated with infected Vero cells.

**Table 2:** Cell-free inactivation of SARS-CoV-2 titres. Titres of SARS-CoV-2 after incubation with selected herbal extracts for the indicated times. Values are expressed as percentage of the titre after treatment with PBS for 1 h (= 100 %)

	15 min	1 h
BRO TP	88 %	62 %
IMU	60 %	41 %
TOP	70 %	35 %

## Discussion

The potential of herbal medicinal products against SARS-CoV-2 and COVID-19 has only sparsely been investigated. Nonetheless, early Chinese guidelines on treatment and control of the newly-emerging pandemic recommended the use of traditional medicines including herbs [24]. One example of an herbal component acting against the virus is micro-RNA extracted from the plant honey suckle, which was described to inhibit SARS-CoV-2 replication [25]. Otherwise, the investigation of herbal metabolites has mostly been limited to *in silico* experiments, using approaches such as molecular docking to assess a potential inhibitory capacity of well-defined plant-derived molecules on viral factors [26–28]. So far, experimental confirmation of anti-viral actions of such herbal metabolites is mostly lacking.

The results of the pilot experiments reported here indicate that some herbal extracts – in particular BRO TP and, to a lesser extent, IMU and TOP – have the potential to interfere with SARS-CoV-2 propagation in Vero cells. The other herbal extracts that were tested (BRO TI and SINx) did not have any apparent effect on SARS-CoV-2 RNA load in this system. Hence, the herbal extracts differed in their potential antiviral capacity in cell culture, suggesting that the effects of the respective extract are specific. Our results do not show equal efficacy for all test items on replication of SARS-CoV2. This confirms the validity of our results due to the complex methodology. The solvent (ethanol) moderately reduced the viral RNA load at the highest concentration under investigation (1:10 dilution of stock, equalling 0.037 % ethanol). This solvent concentration was well above the CC_50_ of each of the extracts under investigation, thus largely excluding that the antiviral action was mediated by the solvent. Direct virus inactivation by the herbal extracts was incomplete upon incubation for 1 h, implying that the antiviral effect of BRO TP was mostly caused by interference with virus replication and/or the cell biology of virus replication. Thus, certain herbal extracts might have the potential to suppress SARS-CoV-2 replication *in vitro*. Herbal medicinal products have already been widely demonstrated to improve the symptomatic burden of respiratory diseases like rhinosinusitis and the common cold [14–16, 29–37]. In addition, these products have been shown to exhibit anti-inflammatory effects which might be beneficial in treating SARS-CoV2 infections [17, 30, 32–36]. The identification of plant-derived molecules could aid in the identification of new lead structures and therapeutic targets for the treatment of SARS-CoV-2 infections in the future. Components of primrose root may be particularly interesting to look at in more detail [31–33]. In independent clinical trials with BRO TI and BRO TP, a comparable efficacy in acute bronchitis was shown [31, 38].

In the cell culture system used for these pilot experiments, toxicity of the extracts was evident at higher concentrations. A likely explanation for this cytotoxicity is the sensitivity of cultured cells to herbal components such as terpenoids and saponins [39]. In the clinical setting however, all tested herbal medicinal products are known to be safe from many years of experience in practical use with only mild or moderate adverse events.

The extracts tested here differ in composition, comprising a range of potential active substances such as flavonoids, terpenoids and polysaccharides. At present, the underlying molecular causes for inhibition of SARS-CoV-2 propagation are unknown. The antiviral activity may be explained by the docking of some of these components to viral proteins, leading to inhibition of virus entry or replication. In addition, some direct virucidal potential is also conceivable, especially for TOP and IMU. To our knowledge, no direct antiviral potential has ever been reported for any of the components of TOP (*Capsicum annuum*, *Guaiacum* and *Phytolacca americana*). Further work is needed to clarify whether the inhibition of viral replication is due to direct interaction with the virus and/or inhibition of a defined viral or cellular protein. Certain metabolites that are contained in the tested herbal extracts such as luteolin, quercitin or apigenin [40] may bind angiotensin-converting enzyme 2 (ACE2, the receptor for SARS-CoV-2) or viral proteins such as the main protease, thus inhibiting virus entry or replication, respectively [26–28]. Since the tested herbal medicinal products comprise a variety of metabolites, it is likely that their components target different steps of viral replication and might act synergistically.

Furthermore, antiviral properties may also be induced by a favourable modulation of the immune response, leading to decreased symptoms. Such immunomodulatory effects are difficult to be assessed in cell culture models. Therefore, an *in vivo* approach is required to further elucidate the complete antiviral potential of herbal medicinal products against SARS-CoV-2. Moderate antiviral effects in cell culture might translate into more pronounced effects *in vivo*.

The most important limitation of the pilot experiments described here is that the results in cell culture are not readily transferable to a clinical situation. Yet, the results provide a first hint that herbal extracts might hold a potential to restrict SARS-CoV-2. All herbal medicinal products assessed in this work have been in use for many years to improve the symptoms of respiratory infections. These products might provide symptomatic relief, particularly in milder cases of COVID-19, which are generally characterised by symptoms resembling those of a common respiratory infection like cough or a sore throat. Herbal medicines might prevent the initial spread of the virus in the upper respiratory tract after exposure to SARS-CoV-2, e.g. in an aerosol, where the viral load is often low. Clearly, further clinical investigations are required and warranted to further elucidate the potential of herbal medicines to target SARS-CoV-2 and to alleviate COVID-19 symptoms.

## Data Availability

The datasets used and/or analysed during the current study are available from the corresponding author on request.

## Abbreviations

ACE2: Angiotensin-converting enzyme 2
BRO TI: Bronchipret^®^thyme-ivy
BRO TP: Bronchipret^®^thyme-primrose
CC_50_: Cytotoxic concentration
COVID-19: Coronavirus disease 2019
DER: Drug–extract ratio
DMEM: Dulbecco’s Modified Eagle’s Medium
IMU: Imupret^®^
MOI: Multiplicity of infection
MTT: 3-(4,5-dimethylthiazol-2-yl)-2,5-diphenyl­tetrazolium bromide
PBS: Phosphate-buffered saline
PFU: Plaque-forming unit
qPCR: Quantitative polymerase chain reaction
RNA: Ribonucleic acid
RSV: Respiratory syncytial virus
SARS-CoV-2: Severe acute respiratory syndrome coronavirus 2
SEM: Standard error of the mean
SINx: Sinupret^®^ extract
TCID_50_: Tissue culture infectious dose
TOP: Tonsipret^®^
